# Advances in Ion Channel, Non-Desmosomal Variants and Autophagic Mechanisms Implicated in Arrhythmogenic Cardiomyopathy

**DOI:** 10.3390/cimb45030141

**Published:** 2023-03-07

**Authors:** Kexin Li, Yufeng Jiang, Yiyao Zeng, Yafeng Zhou

**Affiliations:** 1 Department of Cardiology, Dushu Lake Hospital Affiliated to Soochow University, Suzhou 215000, China; 2 Institution for Hypertension of Soochow University, Suzhou 215000, China

**Keywords:** arrhythmogenic cardiomyopathy, ion channel, autophagy, non-desmosomal variant

## Abstract

Arrhythmogenic cardiomyopathy (ACM) is a heterogeneous disorder characterized by the replacement of cardiac myocytes with fibro-fatty tissues, leading to abnormal excitation-contraction (EC) coupling and a range of malignant events, such as ventricular tachycardia (VT), sudden cardiac death/arrest (SCD/A) and heart failure (HF). The concept of ACM has recently been ex-tended to include right ventricular cardiomyopathy (ARVC), left ventricular cardiomyopathy (ALVC) and biventricular cardiomyopathy. ARVC is generally seen as the most common type of ACM. The pathogenesis of ACM involves mutation variants in desmosomal or non-desmosomal gene loci, as well as various external factors, such as intense exercise, stress and infections. Ion channel alterations, autophagy and non-desmosomal variants are also important components in the development of ACM. As clinical practice enters the era of precision therapy, it is important to review recent studies on these topics to better diagnose and treat the molecular phase of ACM.

## 1. Introduction

With the spread of molecular diagnostics and increased awareness of medical screening, non-ischemic cardiomyopathies are gradually coming into the limelight. Although the incidence of ACM is approximately 1/2000–1/5000 [1], exclusionary diagnostics are often used and the research for the cause of non-ischemic cardiomyopathy is still a foggy task [2]. Disorders of mechanical and electrical function are more regularly encountered in the right ventricular free wall (RVFW) and outflow tract (RVOT), rendering the term ARVC more commonly used in the field. However, phenotypes of ALVC and biventricular (i.e., ACM) have also been reported, so the definition of ACM is now being expanded to include the broader category of ACM [3]. Notably, an expert consensus in the field defines ACM as a disease that encompasses both structural abnormalities (by imaging and pathology) and ventricular arrhythmias [4]. Some controversy exists as to whether ACM should only include inherent arrythmias, such as Brugada Syndrome (BrS), CPVT etc., regardless of the extra environmental cause (e.g., cardiac sarcoidosis). What is explicit is that documented in recent guidelines for practice suggests that genetic testing is a crucial part of the management of these conditions [3]. In addition, most doctors used to think that ACM was different from primary cardiac electrical disease in terms of their pathologies, with primary generally being a pure genetic variant in a subunit of the ion channel and ACM being a genetic variant leading to changes in the desmosomes of cardiomyocytes. Nowadays ACM is seen to be an inherited disease caused by a combination of internal genetic variants, including ion channels, desmosomes, non-desmosomes, autophagy, immune-associated cytokines and other external factors, as the causative gene is not completely penetrant [5,6,7]. Since we are now at the stage of precision therapy where not every patient with ACM will have fixed variants with the discovery of desmoplakin (*DSP*), plakophilin-2 (*PKP2*), desmoglein-2 (*DSG2*), Junction Plakoglobin Catenin (*JUP*), transmembrane protein 43 (*TMEM43*) and desmocollin-2 (*DSC2*) (Table 1), which have been robustly validated as the pathogenic genes for ACM [7,8], but also other variants in non-desmosomal proteins, which range from variants at the molecular level to the appearance of phenotypic defects that result in the loss of cardiomyocytes, the accumulation of inflammatory factors and the combined alteration of the metabolic environment of cardiomyocytes that ultimately leads to the appearance of cardiomyocytes being replaced by fatty-fibrous tissues [2,9]. There is no single diagnostic criterion and no curative treatment for ACM.

Autosomal dominant inheritance predominates in ACM with most patients having pathogenic variants in one or more genes encoding desmosomes. Consequently, it is thought that abnormal desmosome function is the ultimate common pathway in ACM. Of course, there are also several pathogenic genes that cause classic ACM that do not encode desmosomes, in which case most of the variant genes encode proteins that either bind ligands for desmosomes or interfere with the function of the protein due to abnormal desmosome function or vice versa, such as abnormal ion channel function that interferes with desmosome function (such as *SCN5A*). It has recently been discovered that intercalated discs can be involved as a whole. With the recognition of desmosomal pathogenic variants, individuals and families with predominantly left ventricular arrhythmias (VA) and structural abnormalities have been identified, as have patients with non-desmosomal arrhythmia-associated variants with a predominantly left (but also right) or biventricular ACM phenotype. All too often, the first presentation in suspect ACM is SCD/A. Notwithstanding, common features include syncope from fatigue, cardiac dysfunction (reduced ejection fraction (EF) <40%), >500 ventricular premature contractions in 24 h, T-wave reversal in the V1-V6 precordial leads, epsilon waves, ventricular arrhythmias with a left bundle branch block pattern (LBBB) and VT [3,10]. An additional report suggests that morphological changes in the right ventricle under catheter angiography could be of some significance in the diagnosis of ACM [11]. The clinical approach is more likely to be the use of anti-heart failure and anti-arrhythmic drugs after the development of VA and HF [12], the implantation of an ICD to prevent SCD, to reduce the occurrence of electrical storms, such as VA/VT [10], and there is also literature on patients with frequent arrhythmias in ACM who have successfully improved their quality of life with radiofrequency ablation procedures [13,14]. If, unfortunately, the patient reaches the terminal stage of heart failure, it might be appropriate to consider heart transplantation as a treatment option. However, it is drinking poison to quench thirst.

Some studies have reported that mild to moderate exercise can increase the expression of *Cx43*, and thus diminish the development of arrhythmias in cardiomyopathy. Paradoxically, *Cx43* is usually not significantly altered in the mild and moderate stages of the disease and decreases significantly in the end stage [15], but it is unrealistic to ask end-stage patients to do light to moderate exercise in clinical practice. Furthermore, the findings of a clinical study suggest that patients with ACM who have ICD should significantly decrease their physical activity level to minimize the occurrence of VA [16,17]. Even though there are currently laboratory results indicating that exosomes can improve ACM cardiac fibrosis in Dsg-2 mutant mice [18], these are only preclinical studies and the current clinical management of ACM patients is still at the stage of traditional optimization of anti-heart failure, prevention of arrhythmogenesis and other evidence-based drugs to improve the quality of life of patients [19]. *SCN5A* is the gene encoding Nav1.5, while the structure of the complex formed by Nav1.5 in voltage-gated sodium channels (Nav) with the antiarrhythmic drugs flecainide, propafenone and quinidine show the binding site of the antiarrhythmic drug in the centrum [20]. Variants in the *SCN5A* gene were also reported in studies related to the ACM [21]. Variants in the *SCN10A* gene have been reported to contribute to arrhythmias possibly through indirect regulation of the non-classical pathogenic pathway of *SCN5A* [22]. Variants in calcium-activated K+ channels in the endoplasmic reticulum also contribute to the presence of VA, HF in ACM [23]. It has also been reported that defects in the *CDH2* gene lead to conformational alterations in the adhesion protein cdh2 on the z-disc, resulting in the impaired transmission of signals between cells [24]. The current research findings report that the abnormalities of ion channels appear to be of second importance in addition to variants in desmosomal genes [25]. Furthermore, some novel ways of cell-death-like autophagy, ferroptosis and other endoplasmic reticulum stress seem to be the rising stars in ACM [6,26,27]. The recent directions of interest in the research seem to demand further insight into the subcellular structure inside the phospholipid bilayer. We will elaborate and review the mechanisms and fresh discoveries pertaining to ion channel alterations as well as intracellular autophagy and non-desmosome gene variations associated with ACM sequentially from the outside-in perspective aiming to further contribute to the later precision therapy.

## 2. Aberrant Ion Channels on the Membrane of Cardiomyocytes Associated with Arrhythmogenic Cardiomyopathy

### 2.1. Voltage-Gated Sodium (Nav) Channels

Nav channels perform an essential and fundamental role in the transmission of electrical signals [20]. Nav is currently classified in the field of pharmacological research as subtypes 1.1–1.9, of which 1.4 and 1.5 are predominantly found on cardiac myocytes. The Na^+^ currents mediated by Nav1.5 include the peak and late components (INa-P and INa-L) responsible for the rising phase of the cardiomyocyte action potential (AP). Mutant Nav1.5 leads to alterations in peak and late Na currents and is associated with an increasingly widespread congenital arrhythmia. Rapid entry of Na currents (INa) is mediated by Nav1.5 channels and this current contributes mainly to the depolarization of the AP in cardiomyocytes and the His–Purkinje system.

*SCN5A*, the gene encoding Nav1.5, was shown in a joint North American–Netherlands study to carry a rare pathogenic variant in approximately 2% of ACM patients, and it was demonstrated in vitro by patient-derived induced pluripotent stem cell differentiated cardiomyocytes (hiPSC-CM) that *SCN5A* variants can lead to decreased Na channel function in cardiomyocytes, as well as down-regulation of the N-cadherin expression at the intercalated disc junctions, suggesting it may contribute to arrhythmogenic cardiomyopathy through a non-classical mechanism [28]. However, another hiPSC study targeting DSG-deficient *SCN5A* variants in mice showed contrary results to the previous study with no significant decrease in Na current seen [29]. This paradoxical nature between the studies derives from the fact that induced stem cells in vitro are inherently more unstable than tissue cells in vivo, beating spontaneously, and due to their immaturity they are more depolarized at rest than adult ventricular cells, which may affect the dynamics of voltage-gated ion channels and thus alter their own excitability [30]. A study from Japan of 303 patients with BrS, ACM negative for the *SCN5A* variant suggested that two *TCAP* gene mutants c.145G > A: p.E49K (chr17: 37822003) and c.458G > A: p.R153H (chr17: 37822316) lead to structural changes in their transcription products telethonin structural alterations, which in turn lead to structural breakage of the z-disk between cardiomyocytes ultimately causing loss of function of the cardiac Na channel (Nav1.5) [31]. Another study reviewed 96 cases of SCD of unknown origin in Scotland over a 13-year period from 2000 to 2013, of which 50 were clearly cardiomyopathies and 26 were ACMs, the most typical pathogenic variant in ACMs being the PKP2 variant accompanied by non-direct pathogenic variants in DSP and *SCN5A* [32]. This result may suggest that *SCN5A* is not directly pathogenic but is involved in the regulation of the disease process. Taken together, the pathogenic mechanism of *SCN5A* in ACM may arise from upstream regulators leading to loss of z-disc structure and further loss of Na channels, which in turn leads to electrical conduction abnormalities and further accelerates the asynchronous activity of the heart leading to cardiomyocyte necrosis; it is also possible that direct variants in *SCN5A* lead to dysfunction of Na channels and, consequently, necrosis of cardiomyocytes due to asynchronous activities and a series of inflammatory responses. It is also possible that a direct variant in *SCN5A* leads to dysregulation of Na channel function, which in turn leads to myocyte necrosis and a series of inflammatory responses. However, the exact pathogenic mechanism remains to be explored.

In addition, variants in *SCN10A*, the alpha subunit encoding Nav1.8, which is found mainly in nociceptive neurons in the dorsal root ganglia and visceral neurons in the heart, have been shown to be associated with atrial fibrillation and Brugada syndrome [33]. A case-control study of *SCN10A* from Hopkins on 151 North American patients with ACM showed no difference in phenotype between patients with variants in the pontine and non-pontine genes in the case group, and similar variant rates in the control group compared to healthy Caucasians, which would suggest that the *SCN10A* variant is not directly causative of ACM. This suggests that the *SCN10A* variant is not directly responsible for ACM but may act to indirectly influence the clinical phenotype of patients by directly affecting late Na currents, indirectly regulating *SCN5A* transcription and firing frequency to intracardiac neurons (Table 1) [22].

### 2.2. Calcium (Ca) Channel

The transmembrane voltage-gated L-type calcium channel (LTCC) has four subtypes classified by the Cav1 family, of which Cav1.2 and Cav1.3 are predominantly distributed on the cardiomyocyte membrane, with 1.2 dominating and 1.3 being unevenly distributed on the cardiomyocyte. It is well known that the excitatory contractile coupling of cardiomyocytes is mainly dominated by the Ca^2+^-induced Ca^2+^ release (CICR). The LTCC on the cell membrane consists of three subunits outside the core region mainly composed of Cav1.2, the β and the α2δ pairs whose role is to anchor and transmit information and, from these, two subunit helices form S1-6, where S1-4 is anchored on the cell membrane as a structural domain for sensing voltage when depolarization of the cell membrane, when the electrical signal is transmitted, opens the internal Cav1.2 core region, which can be considered the first step in the initiation of the CICR electrical spark [30].

**Figure 1:**
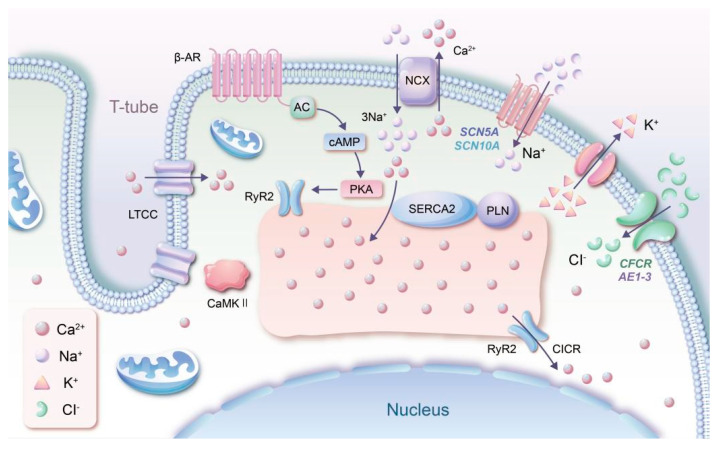
The surface of the cardiomyocyte membrane is recessed to form a T-tube structure on which LTCC calcium ion channels are distributed. The other ion channels are the Na–Ca exchanger NCX, Na channels (*SCN5A*, *SCN10A*), K channels (*KCNN*), Cl channels (*CFCR*, *AE1*-*3*) and the G protein-coupled receptor β-AR. Inside the cells there is a SR/ER that stores Ca^2+^. On the surface of the SR/ER are RyR2, SERCA2, PLN and the cytoplasmic CaMKII calcium-regulated kinase, which together mediate the flow of Ca^2+^ into the SR/ER and its release. The ion channels mentioned in the figure all have papers suggesting that variants of ion channel proteins and related proteins would have an impact on the pathogenesis of ACM. These papers are reviewed in the relevant sections.

Many studies have shown that variants in LTCC channels can lead to abnormal electrical conduction, SCD and other malignant events in ACM patients. The main mechanism of action is that variants in the LTCC gene result in a change in the conformation of the protein and unstable or failed Ca ion binding at the molecular level, leading to abnormal excitability and mechanical activity in cardiomyocytes and ultimately to myocardial pump failure, SCD/A and other malignant events. The altered distribution of LTCC across the cell membrane during disease progression to heart failure is also inseparable from the two kinase activities of calcium–calmodulin (CaM) dependent protein kinase II (CaMKII) and PKA on LTCC. It has been shown by computer 3D simulations that the distribution of LTCC on the cell membrane is significantly different in normal cardiomyocytes, DCM and ICM, with LTCC present on the T-tubules in DCM and the protruding cell membrane surface LTCC changing to phosphorylated LTCC-P and activated by CaMKII in DCM cells, but LTCC-P in ICM is activated by PKA (Figure 1) [34]. It has also been shown that the *BIN1* gene, a membrane scaffolding protein of the BAR domain protein superfamily, is the gene that initiates the invagination of skeletal muscle cell membranes to produce T-tubules, and this gene has also been reported to be associated with the anchoring of Ca1.2 on T-tubules, and knockdown of this gene results in reduced Ca inward flow into the cell membrane and binding to RyR2 resulting in a reduced transient current [35]. Recent studies have reported that Ca ion channels may alter intercellular junctions and intracellular signaling in ACM and thus play an important role in ACM pathogenesis, but most of these studies have been based on the presence of altered desmosomal genes rather than non-desmosomal. Among the non-desmosomes associated proteins related with Ca ion channel regulation is phospholamban (PLN), one of the major regulators of the Ca^2+^ cycle. PLN is sensitive to PKA-dependent phosphorylation and when it is phosphorylated, the inhibitory activity of sarcoplasmic/endoplasmic reticulum (SR/ER) Ca^2+^-ATPase (SERCA) inhibitory activity is abolished and the affinity of SERCA for Ca^2+^ is restored to the extent that large amounts of Ca ions are pumped back into the SR [36]. Removal of phosphorylated PLN interacts with SERCA, resulting in a low Ca^2+^ affinity state that limits Ca^2+^ entry into the SR lumen for storage [37]. Currently, most of the studies on Ca channels are on catecholaminergic polymorphic ventricular tachycardia (CPVT) subjects and rarely in ACM. The principal reason might be that while this channel is closely interconnected with RyR2, it also has some overlaps in pathogenic pathways with inherited arrhythmias, such as CPVT, and its pathogenic mechanisms in ACM could be further explored in the future.

### 2.3. Potassium (K) Channel

Calcium-activated K channels include small conductance (SK1, SK2 and SK3) and intermediate conductance (SK4, also known as KCa3.1) channels. The intermediate conductance calcium-activated K channels (*KCNN4*, SK4) are associated with arrhythmias in atrial fibrillation (AF) and CPVT. SK1-3 channel currents can alter AP-duration (APD), while SK4 channel currents can affect cardiomyocyte pacing activity [23]. Results from a study of SK channels in dogs suggests that SK2 (*KCNN2*) is predominantly distributed in the pulmonary veins rather than the left atrium and that SK channels occur in AF [38]. Results from another multicenter study in humans suggest that the variant chromosome 1q21 to lone AF: rs13376333, which encodes the gene SK3 (*KCNN3*), is significantly associated with the risk of AF in humans, a finding that highlights the potentially important role of SK channels in AF [39]. In a study on CPVT, it was mentioned that SK4 channel blockers protected calsequestrin 2 (CASQ2, the gene predisposing to CPVT), CASQ2-D307H KI and CASQ2 KO mice from deleterious polymorphic ventricular tachycardia (PMVT). Despite the blockade of SK4 channels, the functional redundancy of Ca^2+^-activated K channels may preserve the delicate balance of inward and outward currents required for normal pacing. Along the same lines, recent studies have shown that cardiac SAN arrhythmias induced by silencing HCN4 (If current) or Cav1.3 (L-type Ca^2+^ current) can be relieved by genetic deletion or pharmacological inhibition of GIRK4 channels (IKACh current). Due to their bradycardic and atrioventricular conduction effects, SK4 channel blockers can be beneficial in preventing ventricular tachycardia by prolonging the refractory period [23]. In a related study in the ACM, it was once again shown that SK4 expression is upregulated in ACM patients. In addition, one study found that in the hiPSC-CMs of an ACM patient, expression levels of nucleoside diphosphate kinase B (NDPK-B) and SK4 channels were upregulated, cellular automaticity was increased and the incidence of arrhythmic events was increased. This study suggests that elevated NDPK-B expression, through activation of SK4 channels, would contribute to arrhythmogenesis in ACM and that NDPK-B may be a potential therapeutic point for the treatment of arrhythmias in ACM patients [40]. In addition, SK4 channels have been shown to have an effect on pacemaker excitability in the sinus node SAN, and application of the SK4 channel blocker TRAM-34 strongly inhibited or completely terminated the spontaneous APs of ACM cells. The SK3 (also known as KCa2.3) was first revealed in vascular endothelial cells and was shown to exhibit potent hyperpolarizing effects and diastolic vascular smooth muscle [41]. Another paper identified SK3.1 in the mitochondria of cardiomyocytes and its agonist protected isolated guinea pig cardiomyocytes from ischemia-reperfusion injury, whereas an antagonist of SK3.1 exacerbated IR [42]. Studies at this stage have not yet found a relevance of SK3 to the pathogenesis of ACM.

### 2.4. Chloride (Cl) Channel

Since the study of ion channels came to the attention of scientists, more attention has been focused on the role of cation channels in the transmission of biological information, although anion channels have also been explored but in a much less in-depth and extensive way than cation channels [43]. It is undeniable that although anion channels do not play as important a role as cations in electrical conduction, their role must not be overlooked. As an important component of the anion channel, the main findings were limited to a few years ago and most of these findings were related to heart failure due to myocardial hypertrophy, and there is no conclusive evidence for Cl channels in ACM. It is now known that Cl is a widely distributed anion channel in tissue cells throughout the body, and the subtypes with clear cardiac distribution include cystic fibrosis transmembrane conductance regulator (*CFCR*), Cl channel 2 and maxi Cl channels, anion exchange protein 1 (*AE1*), anion exchange protein 2 (*AE2*), anion exchange protein 3 (*AE3*) and calcium activated Cl channel (Figure 1). The various Cl channels in the heart may contribute to a range of physiological cellular functions (including cell excitability, cell volume homeostasis and apoptosis) and are relevant, mainly in cardiomyocyte repolarization and prolongation of APD, and they may play an important role in arrhythmogenesis, ischemic preconditioning of the heart and adaptive remodeling of cardiac hypertrophy and heart failure [44,45]. They may also play an important role in myocardial hypertrophy and adaptation remodeling. It can only be inferred that variants in Cl channels resulting in altered channel conformation may contribute to arrhythmogenesis, but the exact mechanism of action in ACM remains to be ascertained [43].

## 3. Abnormalities of Ion Channel Proteins on the Endoplasmic/Sarcoplasmic (ER/SR) Reticulum Associated with Arrhythmogenic Cardiomyopathy

### Ryanodine Receptor 2 (RyR2)

In recent years an increasing number of studies have identified a number of novel genes unrelated to desmosome associated with autosomal dominant ACM. *RyR2* has been shown to be associated with stress-induced polymorphic ventricular arrhythmias (PVA) combined with mild right ventricular motility abnormalities. [46]. Variants in this gene can lead to intracellular calcium overload, inducing cardiomyocyte injury or death, which in turn leads to myocardial dysfunction and conduction abnormalities. A study showed that in striated muscle, *RyR2* is located at the endoplasmic reticulum–plasma membrane (ER-PM) junction formed by the SR and T-tubules and that their mode of action may be through phosphorylation by phosphokinase, which increases the sensitivity of the channel, and through the interaction of the CICR of the plasma membrane store-operated Ca^2+^ channel (SOCs), and the Ca^2+^ on the ER are found to exchange information through the Ca^2+^-release-activated Ca^2+^ (CRAC) channel (Figure 1). [47]. It is well established that the major factor in excitatory contractile coupling in the myocardium is generated by the CICR of the voltage-gated L-type Ca^2+^ channel so that in cardiac myocytes *RyR2* interacts with the channel. *RyR2* is thought to be more associated with CPVT. Some argue that myocardial structural abnormalities caused by *RyR2* variants may be due to CPVT rather than the mutated gene that directly causes ACM as its clinical and pathological features do not show a characteristic ACM-like fibrofatty infiltrative changes [48]. In ACM-related studies, a study by Sanger Sequencing found that *RyR2* pathogenic variants occurred in up to 9% of 64 patients with ACM who were clearly non-desmosomal variants and that there was no clinically significant variability in those carrying this variant, which also showed resting electrophysiological abnormalities, right ventricular dilation and motor abnormalities and pathological fibrofatty infiltrates. The results of this study suggest that the *RyR2* variant is associated with ACM and has not been seen to be superimposed on the CPVT phenotype, which is contrary to the previous findings [49]. This result suggests that the *RyR2* variant plays a role in the genetic basis of conventional ACM, either as one of the causative variants or as a modifier gene. However, it is also possible that the conflict between these two views is due to the small sample size of the latter and the fact that other pathogenic genes cannot be excluded from acting together. An article suggested that *RyR2* should be removed from the causative genes for ACM as more clinical data suggest that it belongs to the CPVT-related field [7]. However, in addition to this article, there have been some recent articles suggesting that variants in *RyR2*-associated genes, such as *TNNI3K*, may be associated with ACM [50]. In addition, the gene variants expressed in our manuscript that affect Ca^2+^ release following alterations in Ca^2+^ channels are also relevant to RyR2. We may suggest that *RyR2* variants are not directly involved in the pathogenesis of ACM but may be responsible for ventricular arrhythmias in ACM through Ca^2+^ disturbances. 

## 4. Mechanisms of Autophagy in Arrhythmogenic Cardiomyopathy

The theory of myocardial degeneration (myocardial dystrophy) was first proposed in 1996, based on the acquired progressive cardiomyocyte hypertrophy and fatty and fibrous tissue replacement pathology that characterizes ACM [51]. Experimental studies have confirmed that the key cascade initiator of normal myocardial fibrofatty replacement is myocyte necrosis and that the molecular mechanisms of myocyte necrosis are one of the current hot topics of research [52]. A growing array of in vivo and in vitro studies suggest that variants in desmosomal genes responsible for abnormal intercellular junctions and cytoskeletal and intracellular homeostasis are essential initiators of myocardial cell necrosis, whereas the mechanisms by which non-desmosomal variants trigger cellular necrosis and its subsequent cascade responses remain to be investigated [28]. There are fewer studies related to novel forms of programmed cell death, such as cellular autophagy and ferroptosis in ACM, but preliminary findings have suggested a possible association with the pathogenesis and disease progression of ACM [5].

**Figure 2:**
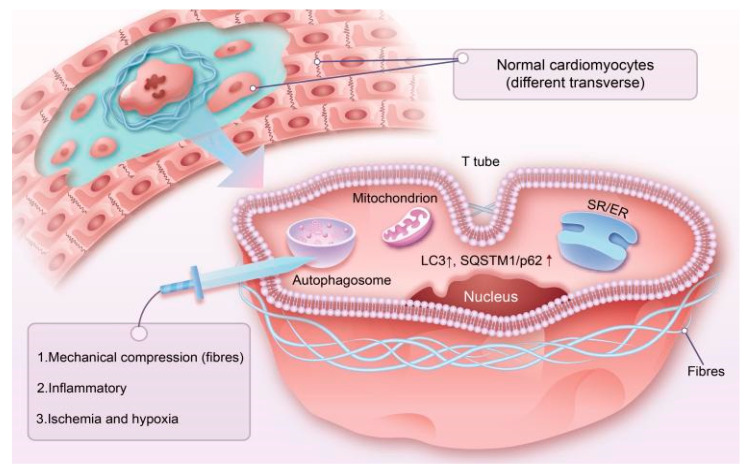
When cardiomyocytes are subjected to long-term injury by external or internal factors (mechanical compression, inflammatory infiltration, ischemia, hypoxia, etc.), cardiomyocytes will gradually perish to form a fibrous encasement around the cardiomyocytes. ACM cells have the pathological process of cardiomyocytes being replaced by fibrofatty tissues, which further creates mechanical compression of normal cardiomyocytes to further aggravate myocardial injury. The internal autophagic mechanism is likely to be the effect of ischemia–hypoxia, a recognized key factor in the initiation of autophagy and heavy mechanical compression of cardiomyocytes in close proximity to the fibrous envelope, further leading to endoplasmic reticulum stress. LC3↑, the gold standard for the detection of autophagy within cardiomyocytes, and SQSTM1/p62↑ suggest that the autophagic pathway is activated or blocked.

Autophagy or autosis, also known as type II programmed cell death, is one of the core functions of all living cells and is a self-regulatory process that involves the destruction of proteins and organelles by cells in response to stimuli from external environmental conditions, such as starvation and stress, while leaving the cell membrane undamaged. Depending on the substrate degraded, autosis is further classified into mitochondrial autophagy, peroxisomal autophagy, lipid autophagy and ferritin autophagy [53]. When autophagy occurs, cells first form single or double membranes, which gradually develop into autophagosomes, which then converge with lysosomes to form autophagic lysosomes using their own hydrolytic enzymes to degrade damaged macromolecules and organelles, thereby recycling the material and maintaining cellular homeostasis. It is widely known that autophagy plays an essential role in the developmental progression and is stimulated by a range of pathogenic factors, such as inflammation and anaerobic conditions and consequent alterations in cellular maturation [54]. It has also been documented that the activation of autophagy reverses the structural remodeling of the myocardium after myocardial infarction, apparently alleviating cardiac dysfunction and producing a corresponding cardioprotective effect [55]. In recent years, attention has been focused on the new field of autosis and ER/SR stress in ACM, and it has been reported that during the development of ACM, overexpression of the autophagic markers LC3 and SQSTM1/p62 was detected in cardiomyocytes next to areas of inflammatory infiltration and fibrosis and containing significant autophagic vacuoles (Figure 2) [6]. Upregulation of Chop mRNA in the right ventricle and reduced expression of RyR2 mRNA also occurred during the chronic disease progression phase of the disease, while ER/SR structures often appeared rapidly enlarged, further demonstrating that altered local autophagy and enhanced ER/SR stress play a role in the pathogenesis of ACM.

Ferroptosis is a novel form of cell death that is dependent on the accumulation of ferric ions leading to lipid hyperoxidation (accumulation of ROS and depletion of polyunsaturated fatty acids) and is morphologically, genetically and metabolically distinct from apoptosis, necrosis, pyroptosis and autophagy, which are types of cell death [56]. The results of several studies have shown that the occurrence of ferroptosis requires the participation of autophagy and that certain factors that regulate cellular autophagy, such as BECN1, Nrf2, STAT3 and p53, also have important functions in the process of ferroptosis, such as the results of a study showing that the accumulation of ROS in ferroptosis requires the occurrence of autophagy and that the inhibition of autophagy attenuates erastin-induced cellular ROS accumulation [57]. However, it is known that autophagy is associated with ACM and there is a correlation between autophagy and ferroptosis, and the involvement of ferroptosis in the pathogenesis of DCM and HCM has been reported in the literature. We speculate that since there are some phenotypic overlaps in ACM, DCM and HCM during disease progression, it is reasonable to assume that ferroptosis may be one of the orientations for future research in ACM.

In the field of cardiomyopathy, it is widely known that cardiomyocytes are permanent cells, and although many medical studies are now tackling the challenge of regenerating cardiomyocytes, they are limited to the field of repairing damage after reperfusion of cardiomyocytes, for instance, the novel development of exosome-related sprays for myocardial infarct injuries can significantly reduce myocardial infarction foci compared to the absence of sprays. However, the complete regeneration of myocytes to their original normal state remains a very challenging task. Most studies related to ferroptosis have now shown that it is one of the ways in which myocytes may suffer during the formation of cardiomyopathy. In addition, proteomic analysis has shown that Heat Shock Protein 70 (HSP70) is significantly increased in patients with chronic heart failure associated with arrhythmogenic right ventricular cardiomyopathy [58] and that inhibitors of the HSP70 family are involved in reversing the inhibition of ferroptosis caused by high expression of dnajc12 through inhibiting phosphorylation of Akt, thereby reversing the resistance of breast cancer cells to chemotherapeutic agents [53]. We can then hypothesize that ferroptosis may play an integral role in the development of ACM. In addition to autophagy and ferroptosis, which are new forms of programmed cell death that have been the subject of much research in recent years, the pathophysiological pathogenesis of ACM suggests that the transformation of cardiomyocytes into adipose and fibrous infiltrates is a process that is of great interest to explore in depth, perhaps in addition to the conformational changes in proteins caused by desmosomal and non-desmosomal genes, including autophagy, which is a different form of cell death than apoptosis, and some intra- and extracellular mechanisms. In addition to the protein conformational changes caused by desmosomes and non-desmosomes, this may also include a different approach to apoptosis, such as autophagy and a combination of proteins, immune mediators and cytokines secreted by extracellular and intracellular vesicles leading to the replacement of cardiomyocytes with adipose and fibrous tissue.

## 5. Novel ACM-Related Genes for Non-Mainstream Cognition

Despite the fact that ACM is considered by most scholars to be a genetic variant, a number of large-scale clinical studies have indicated that there is still a population of ACM patients without a distinct pathogenic variant [59,60,61], which suggests that there are novel pathogenic genes awaiting discovery. In one study, whole exome sequencing (WES) of two cousins with confirmed ACM identified a new variant site in CDH2 (c.686A > C, p. Gln229Pro), and sequencing of suspected ARVC patients who were genetically negative also suggested the identification of an additional possible CDH2 variant (c.1219G > A, p. Asp407Asn) that could be causative. This validates that the CDH2 gene variant may be a novel pathogenic gene for ARVC [24]. The Filamin C (FLNC) gene variant has recently been suggested to be one of the non-desmosomal pathogenic genes in ACM, and its pathogenic mechanism is different from the classical desmosomal gene mutation pathogenesis [62,63]. However, FLNC still requires further clinical data and ACMG evidence to validate its confidence as an ACM pathogenic gene. In addition, a variant in the nuclear envelope protein transmembrane protein 43 (*TMEM43*) gene, a recently noted cell membrane surface protein, has also been shown to be an ACM pathogenic gene with strong evidence [7]. Moreover, the protein obscurin, which is mainly expressed in the SR of cardiomyocytes, as well as its gene *OBSCN*, have been previously suggested to be structural proteins connecting the sarcomere M-line to the SR and to be implicated in striated muscle formation, and the *OBSCN* gene has been associated with hypertrophic cardiomyopathy. However, a study reported for the first time that *OBSCN* variants were associated with the pathogenesis in ARVC and that ARVC-iPSC-CMs exhibited increased intracellular calcium, lipid droplets, increased pleomorphism and irregular Z-bands compared to controls. Differential expression enrichment analysis in the ARVC group indicated that the gene was mainly associated with cell adhesion and structure in the lesion, as well as adipocytokines and PPAR signaling pathways [64]. A study from Japan of 303 BrS, ARVC patients with no *SCN5A* variant suggested that two TCAP gene mutants c.145G > A: p.E49K (chr17: 37822003) and c.458G > A: p.R153H (chr17: 37822316) lead to structural changes in their transcription products telethonin structural alterations, which in turn lead to structural breakage of the z-disk between cardiomyocytes, ultimately causing loss of function of the cardiac Na channel (Nav1.5) [31]. The results of a study at Peking Union Medical College Hospital also suggest that myotonic dystrophy patients with variants in the Duchenne muscular dystrophy (*DMD*) gene can also have a pathological phenotype akin to that of left ventricular ACM with typical fibrofatty infiltrative features [65]. Another multicenter study found that integrin β1D protein expression was distinctly and specifically downregulated in ARVC and further revealed that its mechanism of effect in ARVC is that integrin *β1D* directly regulates RyR2 channel activity by inhibiting channel opening probability and timing to cause disease. Integrin β1 consists of four isoforms, including β1A, B, C and D. Integrin β1D is predominantly expressed in transverse muscle, of which is the only isoform expressed in adult cardiomyocytes [66]. Gene deficiency of integrin β1D predisposes ARVC to VT events. Another study found that GNS panel analysis in two Italian families of ACM found missense variants in the *MYH7* gene to be likely pathogenic in the classification of American College of Medical Genetics and Genomics (ACMG) [67].

This suggests a high degree of genotypic and phenotypic overlap between ACM and DCM under existing diagnostic and disease classification conditions. A previous paper by our team also reported a patient with frequent ventricular tachycardia in IDCM with an echocardial report suggesting a large cardiac LVDD of 90 mm but lacking a biopsy and genetic diagnosis to verify the genotypic overlap with ACM (Table 1) [68].

**Table 1:** Pathogenic and likely pathogenic (LP) genes related to ACM.

	Gene	Credibility	References
Non-desmosomal genes	*TMEM43*	Strong	[7]
	*PLN*	Moderate	[37,69]
	*OBSCN*	Moderate	[64]
	*SCN5A*	Moderate	[21,25]
	*SCN10A*	Moderate	[22]
	*TCAP*	Moderate	[31]
	*TTN*	Moderate	[8]
	*CDH2*	Moderate	[24]
	*TGF-β3*	Moderate	[25]
	*LMNA*	Moderate	[70]
	*BIN1*	Moderate	[71]
	*DMD*	Moderate	[18]
	*Integrin β1D*	Moderate	[66]
	*MYH7*	Moderate	[67]
	*FLNC*	Moderate	[63]
	*TNNI3K*	Moderate	[50]
Desmosomal genes	*JUP*	Strong	[7,52]
	*DSP*	Strong	[7,72]
	*PKP*	Strong	[7,52,73]
	*DSG2*	Srong	[7,59]
	*DSC2*	Strong	[7,59]

Table 1 shows the genes related to ACM mentioned in the manuscript. The credibility of the pathogenic and likely pathogenic (LP) genes in the above tables is based on the reference [7].

## 6. Discussion

It is easy to see that patients often present clinically with complaints of arrhythmias, such as palpitations and syncope, and that frequent premature ventricular beats and ventricular tachycardia may lead to hemodynamic abnormalities and ultimately to changes in the patient’s vital signs, such as blood pressure [74]. The treatment of ACM is also based on the clinical manifestations of the disease. ICDs can prevent SCD and it is necessary to identify patients at risk of SCD in order to decide whether or not to treat them with ICDs. The recommendation for ICD in individuals with ACM is Class I, Level B evidence. The aim of pharmacological therapy in ACM is to control ventricular size and function, manage congestive symptoms and prevent and treat arrhythmias. β blockers are reasonable in individuals with ACM without ICD (Class IIa Level C Evidence). Amiodarone (Class IIb Level B Evidence) and sotalol (Class IIb Level C Evidence) may be reasonable in individuals with ACM for symptom control or to reduce ICD electroshock. Catheter ablation using a combined endocardial/epicardial approach is reasonable in individuals with ACM with recurrent symptomatic sustained VT who have failed to respond to antiarrhythmic drugs or are intolerant (Class IIb Level B evidence) [10]. In addition, the etiology of ACM is under investigation as the rate of penetrate of variants is not significant and there may be clusters of variants in a lineage with different disease phenotypes, which poses a considerable challenge for clinical diagnosis and treatment. We hypothesize that in the pathogenesis of ACM there are not only desmosomal and non-desmosomal genes, but also a defect in a crucial factor in the gene expression process or in the promotion of disease development, which results in a snowball-like cascade with immune inflammatory cell infiltration, replacement of cardiomyocytes by adipocytes and fibrous tissues, which in turn leads to a reduction in cardiomyocytes and thus to a reduction in superficial signaling, such as ion channels and membrane proteins. These mechanisms are interconnected and form a complex modulatory network that leads to the development of ACM. Likewise, owing to the intrinsic relevance of the type and pathogenesis of cardiomyopathies and the degree of overlap in disease phenotypes, variants in these ion channels and channel-associated activator/inhibitor proteins may synergistically lead to arrhythmic episodes in cardiomyocytes, as well as in the pathogenesis of ACM. Although ACM-related ion channel studies are currently scarce and most studies are defined as moderate or supportive within the ACMG criteria, genetic diagnosis and screening are necessary for future clinical precision therapy and may even be aimed at proposing preventive measures for carriers who do not exhibit the disease phenotype. The combination of current research suggests that ACM is not a disease that can emerge as a result of one variant, and its low penetrate and the high heterogeneity of the disease phenotype presented a huge clinical challenge. Moreover, Cx43 is paradoxical in terms of exercise intensity, with studies suggesting that light to moderate exercise induces myocardial output of Cx43 to protect the myocardium of patients with ACM [15,16,17]. Further clinical data and the ACMG evidence chain will be required for new investigations of ion channels and non-desmosomal variants to support these desmosomes from likely pathogenic (LP) to confirmed pathogenic variants, and ion channel and non-desmosomal variants will also be the subject of studies of non-classical pathways of ACM that go beyond the classical pathway of pathogenicity of desomosomal variants in the era of precision medicine.

## 7. Perspectives

In addition to the classic pathogenic pathway of variants in desmosomes, ion channels, linker proteins and nuclei have been implicated in ACM but also in the development of arrhythmic symptoms due to gap junctional remodeling, imbalance of ion homeostasis and connexins. The current amount of research on ion channels has focused on Na channels, with a small number of Ca and K channels and few reports on Cl channels. It has also been shown that ACM can contribute to the development of arrhythmic symptoms by upregulating LC3 and SQSTM1/p62, which are associated with cellular autophagy and endoplasmic reticulum stress. Future research is likely to focus more on non-desmosomal variants. At present, the pathogenic genes of ACM patients are not well studied, and the interactions between the ACM gene regulatory network and the environment still need to be further elucidated.

## Data Availability

Not applicable.
